# Penetration of the SARS-CoV-2 Spike Protein across the Blood–Brain Barrier, as Revealed by a Combination of a Human Cell Culture Model System and Optical Biosensing

**DOI:** 10.3390/biomedicines10010188

**Published:** 2022-01-17

**Authors:** Dániel Petrovszki, Fruzsina R. Walter, Judit P. Vigh, Anna Kocsis, Sándor Valkai, Mária A. Deli, András Dér

**Affiliations:** 1 Institute of Biophysics, Biological Research Centre, ELKH, Temesvári Krt. 62, H-6726 Szeged, Hungary; petrovszki.daniel@brc.hu (D.P.); walter.fruzsina@brc.hu (F.R.W.); vigh.judit@brc.hu (J.P.V.); annabrief@gmail.com (A.K.); valkai.sandor@brc.hu (S.V.); 2 Doctoral School of Multidisciplinary Medical Sciences, University of Szeged, Dóm Tér 9, H-6720 Szeged, Hungary; 3 Doctoral School of Biology, University of Szeged, Közép Fasor 52, H-6726 Szeged, Hungary

**Keywords:** biosensor, Caco-2 cells, coronavirus spike protein, human brain endothelial cell, integrated optics, Mach–Zehnder interferometer, permeability, tissue barriers

## Abstract

Since the outbreak of the global pandemic caused by severe acute respiratory coronavirus 2 (SARS-CoV-2), several clinical aspects of the disease have come into attention. Besides its primary route of infection through the respiratory system, SARS-CoV-2 is known to have neuroinvasive capacity, causing multiple neurological symptoms with increased neuroinflammation and blood–brain barrier (BBB) damage. The viral spike protein disseminates via circulation during infection, and when reaching the brain could possibly cross the BBB, which was demonstrated in mice. Therefore, its medical relevance is of high importance. The aim of this study was to evaluate the barrier penetration of the S1 subunit of spike protein in model systems of human organs highly exposed to the infection. For this purpose, in vitro human BBB and intestinal barrier cell–culture systems were investigated by an optical biosensing method. We found that spike protein crossed the human brain endothelial cell barrier effectively. Additionally, spike protein passage was found in a lower amount for the intestinal barrier cell layer. These observations were corroborated with parallel specific ELISAs. The findings on the BBB model could provide a further basis for studies focusing on the mechanism and consequences of spike protein penetration across the BBB to the brain.

## 1. Introduction

### 1.1. Scientific Background and Purpose of the Study

**Figure 1:**
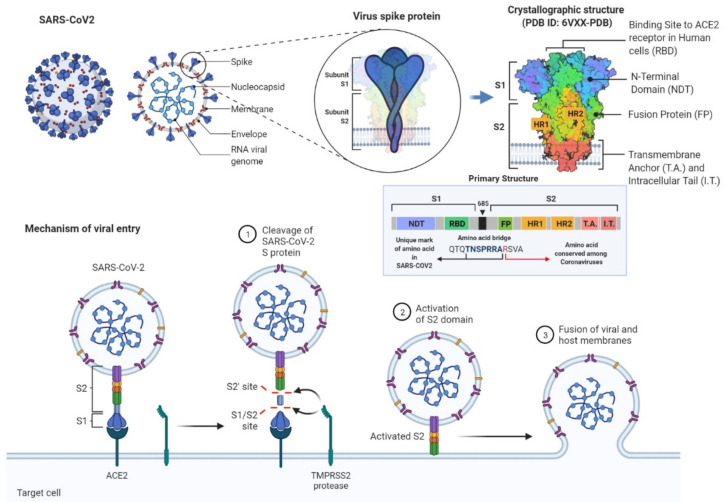
The schematic representation of the SARS-CoV-2 and its surface spike protein structure with their structural descriptions and detailed mechanisms of the viral entry to cells during infection. Spike protein plays a crucial role in this process. While S1 subunit is responsible for anchoring the virion by binding to the cellular receptor angiotensin-converting enzyme 2 (ACE2) of the host cell, S2 subunit enhances the fusion of the viral and the host cell membranes. The fusion is mediated by the S2 subunit that is activated by the transmembrane protease serine 2 (TMPRSS2) cleaving the spike protein at the S1/S2 sites. Adapted from “An In-depth Look into the Structure of the SARS-CoV2 Spike Glycoprotein”, “Human Coronavirus Structure” and “Mechanism of SARS-CoV-2 Viral Entry” by BioRender.com (accessed on 30 November 2021) [3].

At the end of 2019, a novel respiratory coronavirus was reported by Chinese authorities, which we know now as the severe acute respiratory syndrome coronavirus 2 (SARS-CoV-2), causing a global pandemic. Infection occurs primarily by the inhalation of the virus, which can spread through epithelial and endothelial barriers to multiple organs, leading to systemic inflammation [1]. It has been established that the binding of the CoV-2 spike glycoprotein to angiotensin-converting enzyme 2 (ACE2) triggers penetration of the virus into endothelial and epithelial cells [2]. While the S1 subunit of the spike protein is responsible for anchoring the virion by binding to the ACE2 cellular receptor of the host cell, the S2 subunit enhances the fusion of the viral and the host cell membranes. The fusion is mediated by the S2 subunit that is activated by the transmembrane protease serine 2 (TMPRSS2) cleaving the spike protein at the S1/S2 sites (Figure 1).

It is well known that, among other targets, SARS-CoV-2 has a strong neuroinvasive capacity causing multiple neurological symptoms [4] with increased blood–brain barrier (BBB) damage and neuroinflammation [5]. There are some indications, however, that not only the SARS-CoV-2 virus but also its S1 spike glycoprotein alone may be responsible for a major part of these problems [6,7,8,9,10,11,12]. It has been shown that the CoV-2 spike protein or its subunits can be relatively easily detached from the virion, due to enzymatic cleavage [6] or heat stress [7]. As a corollary, S1 spike protein subunits can be detected from the blood, and S1 protein serum levels are associated with the severity of the disease [8]. Moreover, the S1 spike protein can activate platelet aggregation [9], leading to symptoms similar to heparin-induced thrombocytopenia [10]. The Toll-like receptor signaling pathway can be stimulated in macrophages [11], and endothelial function can be impaired via a similar route by the S1 spike protein [12].

Due to the medical relevance and the pathological importance of the S1 spike protein, its detection in the blood stream during infection has come into attention by different methods. It has been shown that the S1 protein of the SARS-CoV-2 crosses the BBB in mice, both when administered intranasally or intravenously [13]; however, the penetration of the S1 spike protein has not been proven on human models of biological barriers. In our present study, we investigated the passage of the SARS-CoV-2 S1 spike protein across cell culture models of the blood–brain and intestinal barriers by using a sensitive optical biosensor system.

### 1.2. Methodological Background

Several highly accurate laboratory methods to detect the virus, and monitor its world-wide propagation, have become a part of the everyday life. By now, people have become acquainted with real-time PCR, as one of the most accurate laboratory techniques to identify the virus even at the early stages of infection in patients [14]. Serological tests have also been widely used in clinical practice during the consecutive waves of the disease.

In order to identify the S1 spike protein being in the focus of the present study, alternative methods are required, based for example on specific antigen–antibody interactions [15]. These techniques are also advantageous to substitute tests requiring laboratory-intensive environment to monitor the spreading of the virus in real-time, even in regions of less-developed healthcare [14,16,17,18]. In such cases, point-of-care diagnostic techniques are preferred to perform rapid, sensitive, and specific detection of the pathogen. Consequently, several biosensor applications as point-of-care diagnostic devices have been developed to provide a good alternative to more complex tests in clinical diagnostics.

In recent years, optical biosensors, more specifically, portable, integrated optical sensor devices, where an optical waveguide is the key element of the detection process, emerged as promising approaches. Most of these devices perform label-free detection, which does not affect their sensitivity significantly but makes them cost-effective. The working principle of such optical biosensors relies on different ways of physical transduction, including interferometry, resonator, or plasmonic techniques [19,20,21]. These sensors translate the capture of an analyte to a measurable change of an optical property, such as refractive index, intensity, or resonance shift, via resonator or interferometric methods. Among optical biosensing platforms, integrated optical Mach–Zehnder interferometer (MZI) is a commonly used interferometric construction, due to its high sensitivity and portability. In this system, the detection is based on the specific binding of the target molecule to the measuring arm of the interferometer made up by a waveguide structure. To reach specificity, the waveguide surface is functionalized by recognition elements, such as receptors, antibodies, or enzymes [21]. Binding of the analyte causes an effective refractive index change in the detection region of the measuring arm, caused by structural changes of the adlayer in the evanescent field of the waveguide. Therefore, a phase difference arises between the propagating waves in the measuring and reference arms, resulting in a change of the output light intensity (For more detail, see Materials and Methods).

### 1.3. Structural Outline of the Paper

The aim of the present study was to investigate and verify whether the SARS-CoV-2 surface S1 spike protein subunit, circulating in the human body during viral infection, could penetrate across tissue barriers directly exposed to the protein. To achieve this goal, the S1 spike protein subunit and in vitro models of the BBB (co-culture of human endothelial cells and pericytes) and the intestinal barrier (Caco-2 cells) were used. To investigate the penetration of S1 proteins across the barrier models permeability assays were performed. To evaluate the amount of spike protein that crossed biological barrier models, an optical biosensing approach was applied. An enzyme-linked immunosorbent assay (ELISA) was used as an alternative detection technique of the target protein validating the results of the optical biosensing technique. After a detailed description of the sensor device, the characterization of the cell culture models is presented, followed by the functionalization of the sensor detection surface, the optical biosensing method, and ELISA immunoassay experiments.

## 2. Materials and Methods

### 2.1. Integrated Optical Mach—Zehnder Interferometer

#### 2.1.1. Fabrication of the Biosensor

The sensor devices used in this study were fabricated and improved based on the construction of the integrated optical MZI interferometer biosensor described in detail in a previous study of our research group [22]. The waveguide structure was made by direct laser writing technique (μPG-101 machine, λ=375 nm, Heidelberg Instruments GmbH, Heidelberg, Germany), as was previously described [23]. To improve the waveguiding performance of the photopolymer structure, the glass substrate was covered with an adhesion promoter layer (Surpass 3000, MicroResist Technology GmbH, Berlin, Germany) by dip coating, following the protocol of the manufacturer. A microfluidic channel was fabricated and integrated in the final form of the sensor device to work with fluid samples [24]. As in our previous study [22], heating was used for setting the bias point prior to the measurements by applying DC voltage on a substrate-bound 20 nm thick gold microheater structure. To improve its adherence to the substrate 10 nm thick Cr was deposited followed by the deposition of the desired gold layer, as was described in one of our latest works [23]. The heater was placed to leave enough space for dissipation, so that the heating of the fluid sample and the measuring arm of the interferometer was avoided, while stabile bias point tuning was achieved.

#### 2.1.2. Biofunctionalization of the Waveguide Structure

To perform specific S1 spike protein detection, the waveguide structure of the interferometer was functionalized in the microchannel. Both interferometer arms, having equal optical length for sensing, were functionalized with antibodies to be capable of specific detection. During the process of the functionalization, the microfluidic channels of the biosensor were filled up by pumping a syringe with a volume of 1 mL (SP210IWZ syringe pump, World Precision Instruments Inc., Sarasota, FL, USA). Firstly, the PDMS microfluidic channels were cleaned with ethanol. Then the microchannels were filled with antibody activation reagent solution of diluted Mix&Go™ at a 1:20 ratio of the stock solution (Mix&Go™ Biosensor, AnteoTech Ltd., Brisbane, Australia) and deionized (18.2 MΩ) MilliQ water (Synergy^®^ UV Water Purification System, Merck-Millipore, Burlington, MA, USA). Incubation lasted for 30 min to enhance the functionalization of waveguide surfaces. Next, the channels were washed by PBS solution (1×, pH = 7.4). The functionalization was performed by filling up the channels with the antibody solution (MonoRab™ SARS-CoV-2 Neutralizing Antibody (BS-R2B2) (Cat. No.: A02051-100), mAb, Rabbit, GenScript, Piscataway, NJ, USA, diluted with 1×PBS at a final concentration of 5 μg/mL) and incubation at 4 °C, overnight. Next day the devices with antibody layers on the waveguide surfaces were washed with a buffer solution (0.1% bovine serum albumin (BSA)–Ringer–HEPES (RH) buffer (150 mM of NaCl, 5.2 mM of KCl, 2.2 mM of CaCl_2_·2H_2_O, 0.2 mM of MgCl_2_·6H_2_O, 6 mM of NaHCO_3_, 2.8 mM of D-glucose, 5 mM of HEPES, pH 7.4)) to remove the unbound antibodies and to match the local refractive index in the vicinity of the waveguides for both arms. After this last step the functionalized sensors became ready to use.

#### 2.1.3. Measurement with the Device

**Figure 2:**
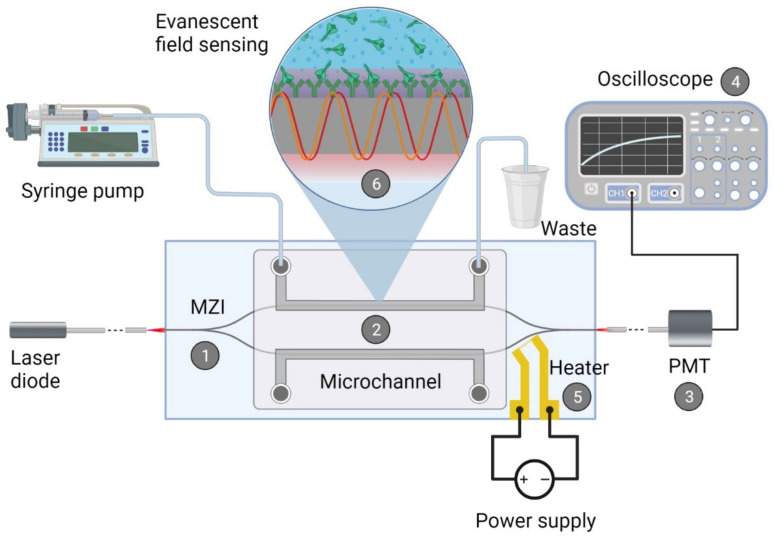
Schematic representation of the biosensor device: the integrated optical Mach–Zehnder interferometer (MZI) for sensing the analyte (1), the microfluidic apparatus (syringe pump, tubes, PDMS microchannel) for fluid sample providing (2), the signal processing unit, namely a photomultiplier tubes (PMT) detector (3) with an oscilloscope (4), the microheater structure for bias point tuning (5). The working principle of the device is also presented: the evanescent field detection is based on the phase difference in the propagating light of the measuring arm (yellow waves) compared to the ones of the reference arm (red waves) (6). Phase difference can be induced by the binding of the target spike protein S1 subunit to the antibody-covered surface of the measuring arm. The figure was created with BioRender.com.

Once the sensor fabrication and functionalization had been completed, the biosensor was prepared for measurements. The construction of the sensor, the techniques of the measuring light coupling, the bias point tuning, and the output signal transmission apparatus were employed from our previous work [13]. As a light source a visible red laser diode (658 nm, 100 mW, RLT650-100MGS, Roithner Lasertechnik GmbH, Vienna, Austria) was used. The continuous flow of the samples was performed the same way as during the functionalization step. The schematic representation of the experimental setup and the integrated optical MZI sensor can be seen in Figure 2.

Prior to the measurements, higher and lower flow rates were applied for filling up the microchannels with the buffer or target protein solutions (30 μL/min and 3 μL/min, respectively). As a starting point of the experiments, the bias point of the integrated optical MZI was adjusted, as has been described previously in detail [22]. To obtain valid detection results by the interferometer, the signal levels and the phase-shifting effect of the microheater at a given applied voltage were set (Figure S1); therefore, output signals obtained from distinct sensors possessing slightly different sensitivities are comparable. The reason behind this lies in the differences in light coupling efficiencies and waveguide structure finesse of such devices. For setting the bias point of the sensor response to the inflexion point on the transmission function, typically 1.0–1.5 V was applied. The adverse effects of bias point drifting on the signal response show the importance of the bias calibration (Figure S2). During this study, the baseline could be kept stable with negligible drift during the measurements on the time scale of several minutes, by using the local heating technique. The results obtained with this experimental setup were analyzed and plotted by MATLAB 2020b software (MathWorks, Natick, MA, USA).

### 2.2. Cell Culture Models and Barrier Integrity Measurements

#### 2.2.1. Cell Culture Models of Biological Barriers

To examine the passage of the spike protein S1 subunit across the BBB we assembled a co-culture model, which consists of human endothelial cells (hEC) and bovine brain pericytes [25,26]. hECs are derived from human umbilical cord blood stem cells and cultured as described previously. Procedure of blood collection was approved by the French Ministry of Higher Education and Research (CODECOH Number DC2011-1321). Informed consent was obtained from the infants’ parents (Béthune Maternity Hospital, Béthune, France). The study was conducted according to the World Medical Association Declaration of Helsinki. hECs arrived at our laboratory at passage number 5 and were grown on 0.2% gelatin (Sigma, part of the Merck Group, Darmstadt, Germany)-coated cell culture dishes (Corning Costar; Corning, NY, USA) and were used at passage number 7 in all experiments. hECs were kept in endothelial cell culture medium (ECM, ScienCell, Carlsbad, CA, USA) supplemented with 5% fetal bovine serum (FBS; Sigma), 1% endothelial cell growth supplement (ECGS, ScienCell) and gentamycin (Sigma, 50 µg/mL). Bovine brain pericytes were cultured on 0.2% gelatin -oated dishes (Corning Costar) in Dulbecco’s modified Eagle’s medium (DMEM, Life Technologies, Thermo Fisher Scientific, Waltham, MA, USA) supplemented with 20% FBS (Sigma), 1% Glutamax (Life Technologies Co., Carlsbad, CA, USA) and gentamicin (50 µg/mL). For both cells when cultures reached confluency, cells were gently trypsinized until cells rounded up, trypsin was removed, and cells were collected and counted in hEC culture medium. To assemble the BBB co-culture model cell culture inserts (Millicell Millipore, MCSP24H48, PET, 3 µm pore size, 24-well format) were coated with a 1:48 ratio of growth factor-reduced Matrigel (Corning) in DMEM in the top compartment (1 h, room temperature). The lower side of the membrane was coated with 0.2% gelatin (20 min, 37 °C). Inserts were dried, and then pericytes were seeded to the bottom of the inserts at a cell number of 5 × 10^3^ cells/insert. Cells were left to adhere for two hours, and then the insert was turned into a 24-well plate (Greiner, Kremsmünster, Austria), and hECs were seeded into the Matrigel-coated top compartment at a cell number of 1.3 × 10^4^ cells/insert. Cells were kept in a co-culture for 4 days while they were fed every 2 days.

To test the passage of the spike protein S1 subunit on an alternative biological barrier, we established an in vitro model of the intestinal barrier using the human Caco-2 intestinal epithelial cell line (ATCC cat. No. HTB-37; Manassas, VA, USA). Caco-2 cells were cultured in DMEM with stable glutamine (Life Technologies) supplemented with 10% FBS (PAN-Biotech GmbH, Aidenbach, Germany) and gentamicin (50 µg/mL). All plastic culture surfaces were coated with 0.05% collagen type I in sterile distilled water. To establish the epithelial barrier model cell culture inserts (Millicell Millipore, MCSP24H48, PET, 3 µm pore size, 24-well format) were coated with collagen type I, and 3 × 10^4^ Caco-2 cells/insert were seeded. Cells were kept on the inserts for 10–12 days and were fed every two days.

#### 2.2.2. Transendothelial Electrical Resistance Measurement

To follow the development of barrier properties on both in vitro models, transendothelial electrical resistance (TEER) was registered every second day before medium change. Plates containing the cells were kept on a heating pad set to 37 °C during the measurement to avoid any fluctuation in the values. For the TEER measurement an EVOM Volt/Ohm Meter with a 24-well cup chamber setup was used (ENDOHM-6G; World Precision Instruments, Sarasota, FL, USA). TEER values were calculated with the subtraction of the background of cell-free inserts (60 Ω cm^2^) relative to the surface of the cell culture insert (0.33 cm^2^) and corrected to the shunt resistance of the insert as described before [27]. TEER values were therefore expressed in (Ω cm^2^).

### 2.3. Spike Protein S1 Subunit Permeability Assays

To examine whether the SARS-CoV-2 spike protein S1 subunit (Genscript, Z03501-100, Piscataway, NJ, USA) crosses the BBB and the intestinal epithelial barrier we established these two models on cell culture inserts as described above. When the TEER reached 34.6 ± 1.9 Ω cm^2^ for the BBB model and 367.76 ± 36.8 Ω cm^2^ for the Caco-2 model, cells were used in the experiments. Passage of spike protein from the top compartment to the bottom compartment of the cell culture inserts was performed in a 0.1% BSA-RH buffer. BSA was present in all samples to avoid attachment of the spike protein to the plastic surfaces. Microcentrifuge tubes and plate wells were also pre-incubated with 0.1% BSA-RH to avoid attachment of the spike protein. During the experiment low-bind pipette tips were used. SARS-CoV-2 spike protein was added to the top compartment of the cell culture inserts (200 µg/mL, 70 µL), while the bottom compartment contained 530 µL low-bind pipette tips buffer. To minimize any unstirred water layer on the surface of the cell layers the plates were kept on a horizontal shaker (150 rpm, Biosan, Riga, Latvia) during the 30 min permeability period. Control cells received only 0.1% BSA-RH buffer. The passage of the SARS-CoV-2 spike protein S1 subunit was also tested on cell-free inserts. Directly after the assay TEER was measured on all inserts and samples were collected from both the top and the bottom compartments and used for the biosensing and ELISA experiments. Permeability assay with fluorescent markers to verify barrier integrity was also performed on control cultures (for protocol and results see Supplementary Material).

### 2.4. Determination of the Passage of SARS-CoV-2 Spike Protein S1 Subunit across Biological Barriers with Elisa

In addition to the biosensing method we validated the passage of the spike protein S1 subunit across the BBB and the intestinal barrier with a traditional method. For this we developed an ELISA, similarly as described previously [28]. All steps were performed at room temperature unless otherwise indicated. Between most incubations a washing step with a wash buffer (TBS buffer and BSA, 10 mM of TRIS-HCl, 150 mM of NaCl and 0.5% BSA at pH = 7.4) was performed. First Nunc MaxiSorp flat-bottom plates (Life Technologies) were pre-treated with a 10% AnteoBind Biosensor solution (AnteoTech Ltd., Brisbane, Australia) for 30 min, then this solution was removed, and plates were dried under laminar flow. Dilution series from the SARS-CoV-2 spike protein stock was prepared between concentrations of 0–20 µg/mL in carbonate buffer (45.3 mM of NaHCO_3_ and 18.2 mM of Na_2_CO_3_ in distilled water, pH = 9.6). Besides the dilution series, samples from the bottom compartments of spike protein treated and control inserts (50 µL/well) were also added to the plate in triplicates and were incubated overnight at 4 °C. The next day, wells were blocked with 1% BSA-5% normal goat serum in TBS buffer for 2 h. After this step the primary antibody (MonoRab SARS-CoV-2 Neutralizing Antibody (BS-R2B2), 1 µg/mL, Genscript, A02051) was added to all wells and was incubated for 2 h followed by the biotin conjugated goat anti-rabbit secondary antibody (0.3 µg/mL, Vector Laboratories Ltd., Burlingame, CA, USA, antibody ID: AB_2313606) for 1 h. To finish the ELISA extravidin peroxidase (0.5 µg/mL, Vector Laboratories Ltd.) was added to each well for 30 min. ELISA substrate tablets containing o-phenylenediamine dihydrochloride (OPD, Life Technologies) were dissolved in 10 mL of citrate buffer (63 mM of Na_2_HPO_4_·2H_2_O, 26.6 mM of citric acid in distilled water, pH = 6) and mixed with 3% H_2_O_2_ solution (25 µL). The substrate solution was pipetted to all wells in a 100 µL/well volume. Yellow color developed within 10 min. The reaction was stopped with 4 N H_2_SO_4_ (50 µL/well). Absorbance was detected with a multiwell plate reader (Fluostar Optima, BMG Labtech, Ortenberg, Germany) at 492 nm. Dilution series data were plotted, and spike protein concentration of each sample was determined based on the calibration curve of the assay (Figure S3).

### 2.5. Statistical Analysis

Data were plotted and analyzed using Excel software (Microsoft, Redmond, WA, USA) and GraphPad Prism 5.0 Software (GraphPad, San Diego, CA, USA). Data are presented as means ± SEM, where relevant. Experiments were repeated at least twice.

## 3. Results

### 3.1. Measurement of the Passage of the SARS-CoV-2 Spike Protein S1 Subunit across Biological Barriers

#### 3.1.1. Integrated Optical Interferometric Biosensor

**Figure 3:**
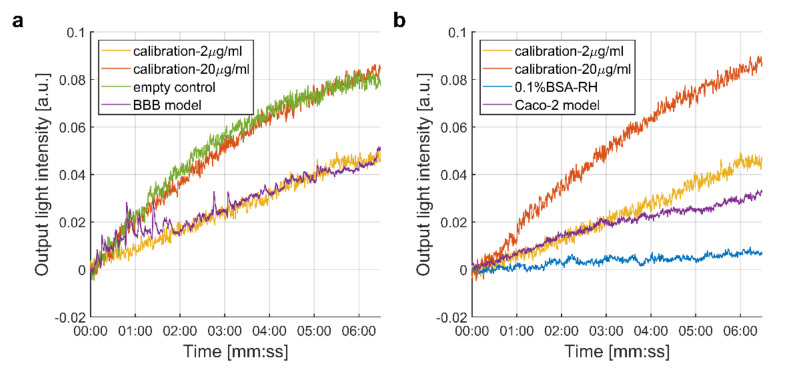
Results of the interferometric biosensing by the integrated optical MZI sensor. Both graphs show the signals detected after the introduction of the calibration samples (2 and 20 µg/mL). (**a**) Results obtained from samples after the passage of the spike protein S1 subunit across cell-free cell culture inserts and the BBB model. (**b**) Signals detected from buffer (0.1% BSA-RH) or samples after the permeability of the spike protein through the Caco-2 monolayer.

The biosensor system was calibrated with control samples of two characteristic spike protein concentrations (2 and 20 µg/mL in 0.1% BSA-RH buffer, Figure 3). For the detection of the target spike protein S1 subunit, samples from the bottom compartments of the permeability assays across the BBB and Caco-2 models were introduced into the measuring arm of the MZI sensor. The upper or the lower arm of the interferometer was referred to as the measuring arm, while the other one remained intact. After each measurement, the corresponding microchannel was leached out with the buffer and could serve as a reference arm for further measurements with the same sensor. Note that as an intrinsic feature of such interferometric detection, the sign of the obtained signal depended on which branch of the interferometer was used as a measuring arm (Figure S4). For the sake of easier comparison, signals are always shown with positive sign. Figure 3 shows the results obtained from the detection of the different solutions: 0.1% BSA-RH buffer, calibration solutions, and samples from permeability assays on both barrier cell culture models and cell-free inserts.

The control buffer solution lacking the target protein (Figure 3b: 0.1% BSA-RH) evoked no significant change in the output intensity, and these signals were measured as a background. Calibration samples with 2 and 20 μg/mL protein concentrations showed suitable signals to compare them to samples obtained from the permeability assays (Figure 3b). In case of the cell-free cell culture inserts (Figure 3a: empty control), we observed a similar signal amplitude as for the calibration sample 20 µg/mL. The evaluated protein concentrations of the target solutions, on the other hand, were different for the two barrier models. In case of the BBB model, the signal obtained with the biosensor was close to the signal of the 2 μg/mL calibration concentration; however, in the case of the Caco-2 layer, the signal was lower, in line with findings of the control ELISA experiments (see under Section 3.1.2).

#### 3.1.2. ELISA Experiments

To validate the results obtained by the biosensing method, we also developed a more traditional ELISA for the detection of the spike protein S1 subunit crossing the BBB and intestinal barrier models. After the spike protein passage assay, TEER was measured, and samples from the bottom compartments of all inserts were collected and used in the biosensing measurements and for the ELISA.

**Figure 4:**
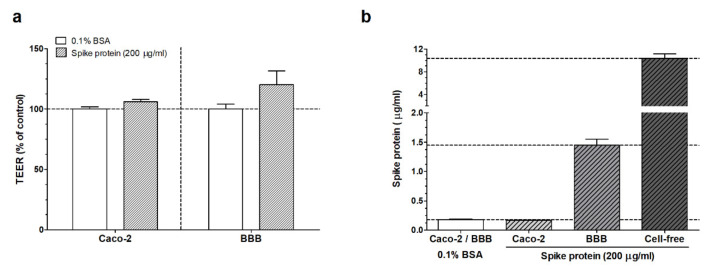
(**a**) Results of the transendothelial–transepithelial electrical resistance (TEER) measurement after SARS-CoV-2 spike protein (200 µg/mL) treatment in 0.1% bovine serum albumin (BSA)–Ringer–HEPES buffer or only after buffer treatment (0.1% BSA). (**b**) Detection of spike protein S1 subunit by ELISA in samples from the bottom compartment of cell culture inserts after spike protein treatment (200 µg/mL, 30 min). We also indicate results for inserts, which received only the carrier buffer (0.1% BSA) or the results of passage of spike protein across cell-free inserts. Caco-2: human intestinal epithelial cells. BBB: blood–brain barrier model.

First, we could conclude that the treatment with 200 µg/mL of SARS-CoV-2 spike protein did not change the TEER of the cultures compared to the control group, which only received the 0.1% BSA-RH buffer (Figure 4a). Permeability for fluorescent markers across control groups also showed a tight barrier for both models (Figure S5). We found, that during our measurements the spike protein S1 subunit crossed cell-free inserts effectively. In the ELISA, spike protein passage was found for brain endothelial monolayers, while the signal measured for the intestinal barrier system was under the detection limit (Figure 4b). These results corroborate the findings of the MZI measurements.

## 4. Discussion

In this study, we investigated the penetration of the well-known surface spike protein S1 subunit of the worldwide pandemic causing SARS-CoV-2 coronavirus through the BBB and the intestinal epithelial barrier systems. The detection of the target spike S1 protein was performed with a portable optical biosensor with rapid interferometric detection as a sensitive technique using an integrated optical Mach–Zehnder device. The results were validated with a traditional ELISA measurement.

### 4.1. Interaction of SARS-CoV-2 and Spike Protein S1 Subunit with Biological Barriers

Here we investigated the passage of the SARS-CoV-2 spike protein S1 subunit across a human brain endothelial cell-based culture BBB model for the first time. Previously it was shown that spike protein treatment alters barrier integrity and induces the activation of endothelial cells [29,30]. During our investigation short-term spike protein S1 subunit treatment did not change the resistance of the BBB model.

The mechanism of virus or viral protein passage across the BBB or epithelial barriers has been studied before and therefore was not in the focus of the current study. Recently it has been found that SARS-CoV-2 crosses mouse and hamster primary brain endothelial cell culture models by a transcellular pathway without changing tight junction expression [31]. It was suggested that S1 subunit crosses the BBB via adsorptive-mediated transcytosis in mice [13], whereas ACE2 receptors were also found to be important in brain uptake in mice [13]. We previously found that the hECs used in the present model [25] express ACE2 only in a very low amount [26]. An extensive review of the SARS-CoV-2 virus and spike protein interactions and alternative passage mechanisms across the BBB was published [32].

Since gastrointestinal symptoms are also observed in COVID-19 we introduced an intestinal barrier culture system to our experimental setup. We found that TEER had not changed after short-term treatment with the spike protein in this epithelial model. In the biosensor measurement we detected the passage of the spike protein across this barrier, which was lower compared to the BBB model’s permeability. Previously it was shown that the Caco-2 cells express the ACE2 receptor, which makes them an appropriate model to study the spike protein passage [33]. The observed difference in the spike protein S1 subunit passage for this culture model could be due to, on the one hand, the basal TEER values, and on the other hand, the distinct receptor expression and alternative routes of passage in epithelial cells. Our study may provide a deeper insight in the barrier function alterations and a quantitative verification of the spike protein penetration across the biological barriers.

### 4.2. Integrated Optical MZI Biosensor

The application of the optical biosensor construction as a sensitive, rapid, and portable way of detecting the target spike protein from fluid samples is one of the strengths of this study. Investigations focusing on the early, effective detection of the virus and its antigens have been in the center of attention [34]. By this label-free, cost-effective approach, the evaluation of the spike protein S1 subunit concentration could be achieved within few minutes. This biosensing approach allowed the investigation of human cell culture model systems of biological barriers. To evaluate the accurate spike protein concentration of the fluid samples, we assumed that the response function of our biosensor followed the Freundlich isotherm, a typical equation valid for a wide range of physical adsorption processes from solutions [35]:

(1) $$log\left(m_{ad}\right)=K+\left(1/n\right)\cdot logC$$

where $m_{ad}$ and C are the relative adsorbed mass and the bulk concentration of the analyte, while K  and n are constants at the given temperature. From the calibration curves measured at the preadjusted concentrations, K=0.3653 and n=3.3219 were obtained, if $m_{ad}$ and C are measured in units given in Figure 3. Using these calibration values, the concentration of the spike protein S1 subunit that crossed the intestinal barrier (Caco-2 cells) was found to be approximately 0.5 μg/mL.

These results show the applicability of this optical biosensing technique for the detection of SARS-CoV-2 surface spike proteins. Such an interferometric, sensing approach is also represented in an ongoing, EU-funded (European Commission), large research project (CoNVat) tackling the COVID-19 pandemic. In that project, a silicone bimodal interferometric sensor is being employed and developed for the detection of the intact virus from nasopharyngeal and saliva samples, as a point-of-care testing device [21,36]. Moreover, various silicone interferometric biosensors have been used for diagnostic purposes for small-molecule [37], protein, glycolipid [38] and nucleic acid detection in the aM-fM range [21,39].

Our results and the above-mentioned studies clearly implicate that integrated optical biosensor platforms have the capability of sensing the spike protein in a scenario of human biological barrier penetration studies. The investigation of BBB models is crucial to understand the neuroinvading consequence of spike protein exposure, the development of neurological anomalies [29]. Therefore, these cell culture studies could be useful model systems for further experiments aiming at the mechanisms of spike protein-associated pathologies.

### 4.3. ELISA

The results obtained by the biosensor measurements were also validated by the ELISA. We observed that spike protein S1 subunit concentrations detected by the ELISA were very similar to the biosensor measurements in the case of the BBB model. This corroborates the results we obtained with both methods. The spike protein passage across the Caco-2 intestinal epithelial cell monolayer was in the detection limit range during the MZI measurements, but it was not detectable by the ELISA. We hypothesize that this occurred due to the different sensitivity of the methods, and we reached the lower detection limit in the ELISA earlier than in the biosensor device. This sensitivity difference was also visible close to the upper detection limit when we measured the passage of the spike protein across the cell-free cell culture inserts.

## 5. Conclusions

This study was focused on a basic evaluation of the barrier penetration capability of the SARS-CoV-2 surface spike protein S1 subunit across two barrier model systems of human organs highly exposed to coronavirus infection. Cell culture systems of the BBB and the intestinal barriers were investigated in this context. Based on the results of the optical biosensing and immunoassay approaches, it was established that spike protein S1 subunit could cross both cell culture models of the barriers but in different amounts. These results and the established models and methods could be valuable for further studies on the pathological effects of SARS-CoV-2 related to the human body.

## Data Availability

The original data are available upon request from the corresponding author.

## Supplementary Materials

The following are available online at https://www.mdpi.com/article/10.3390/biomedicines10010188/s1, Methods: description of barrier integrity permeability studies. Figure S1: The demonstration of the bias point calibration process with the Mach–Zehnder interferometer (MZI) output intensity values during the biasing process and during the measurement. Figure S2: Sinusoidal transmission function curve of the Mach–Zehnder interferometer with the highlighted, ideal inflexion points for biasing on it in a function of the applied voltage, applied for the thermal phase shifting ($U_{\pi /2}$, $U_{\pi }$, $U_{3\pi /2}$ etc.). Figure S3: ELISA calibration. Figure S4: Demonstration of the intrinsic feature of such interferometric detection. Figure S5: Permeability of the Caco-2 intestinal barrier culture monolayer and the human endothelial cell (hEC)-pericyte blood–brain barrier co-culture model.

## References

[1] Gustafson D., Raju S., Wu R., Ching C., Veitch S., Rathnakumar K., Boudreau E., Howe K.L., Fish J.E. (2020). Overcoming barriers: The endothelium as a linchpin of coronavirus disease 2019 pathogenesis?. Arterioscler. Thromb. Vasc. Biol..

[2] Zhou P., Yang X.L., Wang X.G., Hu B., Zhang L., Zhang W., Si H.R., Zhu Y., Li B., Huang C.L. (2020). A pneumonia outbreak associated with a new coronavirus of probable bat origin. Nature.

[3] Biorender.com. Templates. https://app.biorender.com/biorender-templates.

[4] De Sousa A.K., de Aguiar Magalhães D., dos Santos Ferreira J., dos Reis Barbosa A.L. (2020). SARS-CoV-2-mediated encephalitis: Role of AT2R receptors in the blood-brain barrier. Med. Hypotheses.

[5] Schwabenland M., Salié H., Tanevski J., Killmer S., Lago M.S., Schlaak A.E., Mayer L., Matschke J., Püschel K., Fitzek A. (2021). Deep spatial profiling of human COVID-19 brains reveals neuroinflammation with distinct microanatomical microglia-T-cell interactions. Immunity.

[6] Ogata A.F., Cheng C.-A., Desjardins M., Senussi Y., Sherman A.C., Powell M., Novack L., Von S., Li X., Baden L.R. (2021). Circulating Severe Acute Respiratory Syndrome Coronavirus 2 (SARS-CoV-2) Vaccine Antigen Detected in the Plasma of mRNA-1273 Vaccine Recipients. Clin. Infect. Dis..

[7] Kiss B., Kis Z., Pályi B., Kellermayer M.S.Z. (2021). Topography, Spike Dynamics, and Nanomechanics of Individual Native SARS-CoV-2 Virions. Nano Lett..

[8] Ogata A.F., Maley A.M., Wu C., Gilboa T., Norman M., Lazarovits R., Mao C.-P., Newton G., Chang M., Nguyen K. (2020). Ultra-Sensitive Serial Profiling of SARS-CoV-2 Antigens and Antibodies in Plasma to Understand Disease Progression in COVID-19 Patients with Severe Disease. Clin. Chem..

[9] Zhang S., Liu Y., Wang X., Yang L., Li H., Wang Y., Liu M., Zhao X., Xie Y., Yang Y. (2020). SARS-CoV-2 binds platelet ACE2 to enhance thrombosis in COVID-19. J. Hematol. Oncol..

[10] Greinacher A., Thiele T., Warkentin T.E., Weisser K. (2021). A Prothrombotic Thrombocytopenic Disorder Resembling Heparin-Induced Thrombocytopenia Following Coronavirus-19 Vaccination. Res. Sq..

[11] Shirato K., Kizaki T. (2021). SARS-CoV-2 spike protein S1 subunit induces pro-inflammatory responses via toll-like receptor 4 signaling in murine and human macrophages. Heliyon.

[12] Lei Y., Zhang J., Schiavon C.R., He M., Chen L., Shen H., Zhang Y., Yin Q., Cho Y., Andrade L. (2021). SARS-CoV-2 Spike Protein Impairs Endothelial Function via Downregulation of ACE 2. Circ. Res..

[13] Rhea E.M., Logsdon A.F., Hansen K.M., Williams L.M., Reed M.J., Baumann K.K., Holden S.J., Raber J., Banks W.A., Erickson M.A. (2021). The S1 protein of SARS-CoV-2 crosses the blood–brain barrier in mice. Nat. Neurosci..

[14] Zhong J.F., Weiner L.P., Burke K., Taylor C.R. (2007). Viral RNA extraction for in-the-field analysis. J. Virol. Methods.

[15] Asghari A., Wang C., Yoo K.M., Rostamian A., Xu X., Shin J.-D., Dalir H., Chen R.T. (2021). Fast, accurate, point-of-care COVID-19 pandemic diagnosis enabled through advanced lab-on-chip optical biosensors: Opportunities and challenges. Appl. Phys. Rev..

[16] Kaltenboeck B., Wang C. (2005). Advances in Real-Time PCR: Application to Clinical Laboratory Diagnostics. Advances in Clinical Chemistry.

[17] Nagasse-Sugahara T.K., Kisielius J.J., Ueda-Ito M., Curti S.P., Figueiredo C.A., Cruz Á.S., Silva M.M.J., Ramos C.H., Silva M.C.C., Sakurai T. (2004). Human vaccinia-like virus outbreaks in São Paulo and Goiás States, Brazil: Virus detection, isolation and identification. Rev. Inst. Med. Trop. Sao Paulo.

[18] Gibbs S.E.J., Ellis A.E., Mead D.G., Allison A.B., Moulton J.K., Howerth E.W., Stallknecht D.E. (2005). West Nile virus detection in the organs of naturally infected blue jays (*Cyanocitta cristata*). J. Wildl. Dis..

[19] Cheung A.Y., Wu H. (2004). Overexpression of an Arabidopsis Formin Stimulates Supernumerary Actin Cable Formation from Pollen Tube Cell Membrane. Plant Cell.

[20] Nguyen H.H., Park J., Kang S., Kim M. (2015). Surface plasmon resonance: A versatile technique for biosensor applications. Sensors.

[21] Soler M., Estevez M.C., Cardenosa-Rubio M., Astua A., Lechuga L.M. (2020). How Nanophotonic Label-Free Biosensors Can Contribute to Rapid and Massive Diagnostics of Respiratory Virus Infections: COVID-19 Case. ACS Sens..

[22] Petrovszki D., Krekic S., Valkai S., Heiner Z., Dér A. (2021). All-Optical Switching Demonstrated with Photoactive Yellow Protein Films. Biosensors.

[23] Petrovszki D., Valkai S., Gora E., Tanner M., Bányai A., Fürjes P., Dér A. (2021). An integrated electro-optical biosensor system for rapid, low-cost detection of bacteria. Microelectron. Eng..

[24] Mathesz A., Valkai S., Újvárosy A., Aekbote B., Sipos O., Stercz B., Kocsis B., Szabó D., Dér A. (2015). Integrated optical biosensor for rapid detection of bacteria. Optofluid. Microfluid. Nanofluid..

[25] Cecchelli R., Aday S., Sevin E., Almeida C., Culot M., Dehouck L., Coisne C., Engelhardt B., Dehouck M.P., Ferreira L. (2014). A stable and reproducible human blood-brain barrier model derived from hematopoietic stem cells. PLoS ONE.

[26] Santa-Maria A.R., Walter F.R., Figueiredo R., Kincses A., Vigh J.P., Heymans M., Culot M., Winter P., Gosselet F., Dér A. (2021). Flow induces barrier and glycocalyx-related genes and negative surface charge in a lab-on-a-chip human blood-brain barrier model. J. Cereb. Blood Flow Metab..

[27] Vigh J.P., Kincses A., Ozgür B., Walter F.R., Santa-Maria A.R., Valkai S., Vastag M., Neuhaus W., Brodin B., Dér A. (2021). Transendothelial Electrical Resistance Measurement across the Blood–Brain Barrier: A Critical Review of Methods. Micromachines.

[28] Sipos E., Kurunczi A., Fehér A., Penke Z., Fülöp L., Kasza Á., Horváth J., Horvát S., Veszelka S., Balogh G. (2010). Intranasal delivery of human β-amyloid peptide in rats: Effective brain targeting. Cell. Mol. Neurobiol..

[29] Buzhdygan T.P., DeOre B.J., Baldwin-Leclair A., Bullock T.A., McGary H.M., Khan J.A., Razmpour R., Hale J.F., Galie P.A., Potula R. (2020). The SARS-CoV-2 spike protein alters barrier function in 2D static and 3D microfluidic in-vitro models of the human blood–brain barrier. Neurobiol. Dis..

[30] DeOre B.J., Tran K.A., Andrews A.M., Ramirez S.H., Galie P.A. (2021). SARS-CoV-2 Spike Protein Disrupts Blood–Brain Barrier Integrity via RhoA Activation. J. Neuroimmune Pharmacol..

[31] Zhang L., Zhou L., Bao L., Liu J., Zhu H., Lv Q., Liu R., Chen W., Tong W., Wei Q. (2021). SARS-CoV-2 crosses the blood–brain barrier accompanied with basement membrane disruption without tight junctions alteration. Signal Transduct. Target. Ther..

[32] Erickson M.A., Rhea E.M., Knopp R.C., Banks W.A. (2021). Interactions of SARS-CoV-2 with the Blood–Brain Barrier. Int. J. Mol. Sci..

[33] Knyazev E., Nersisyan S., Tonevitsky A. (2021). Endocytosis and Transcytosis of SARS-CoV-2 across the Intestinal Epithelium and Other Tissue Barriers. Front. Immunol..

[34] Singh V., Allawadhi P., Khurana A., Banothu A.K., Bharani K.K. (2021). Critical neurological features of COVID-19: Role of imaging methods and biosensors for effective diagnosis. Sens. Int..

[35] Freundlich H. (1906). Uber die adsorption in Losungen. Z. Phys. Chem..

[36] Combating 2019-nCoV: Advanced Nanobiosensing Platforms for POC Global Diagnostics and Surveillance|CoNVat Project|H2020|CORDIS|European Commission. https://cordis.europa.eu/project/id/101003544.

[37] Chocarro-Ruiz B., Fernández-Gavela A., Herranz S., Lechuga L.M. (2017). Nanophotonic label-free biosensors for environmental monitoring. Curr. Opin. Biotechnol..

[38] Ramirez-Priego P., Martens D., Elamin A.A., Soetaert P., Van Roy W., Vos R., Anton B., Bockstaele R., Becker H., Singh M. (2018). Label-Free and Real-Time Detection of Tuberculosis in Human Urine Samples Using a Nanophotonic Point-of-Care Platform. ACS Sens..

[39] Huertas C.S., Fariña D., Lechuga L.M. (2016). Direct and Label-Free Quantification of Micro-RNA-181a at Attomolar Level in Complex Media Using a Nanophotonic Biosensor. ACS Sens..

