# Supplementary material for: Penetration of the SARS-CoV-2 Spike Protein across the Blood–Brain Barrier, as Revealed by a Combination of a Human Cell Culture Model System and Optical Biosensing
Source: Biomedicines. 2022 Jan 17;10(1):188. doi: 10.3390/biomedicines10010188 (PMC8773803; doi:10.3390/biomedicines10010188)
Supplement: Supplementary file 1 [file biomedicines-10-00188-s001.zip › biomedicines-1511886-supplementary.pdf]

Supplementary information

Penetration of the SARS-CoV-2 spike protein across the blood-brain barrier, as revealed by a combination of a human cell culture model system and optical biosensing

Dániel Petrovszki [1,2,#], Fruzsina R. Walter [1,#], Judit P. Vigh [1,3], Anna Kocsis [1], Sándor Valkai [1], Mária A. Deli [1,#], András Dér [1,#,*]

[1] Institute of Biophysics, Biological Research Centre, ELKH; Temesvari krt. 62, Szeged, H-6726, Hungary; petrovszki.daniel@brc.hu (D.P.); walter.fruzsina@brc.hu (F.R.W.); vigh.judit@brc.hu (J.P.V.); annabrief@gmail.com (A.K.); valkai.sandor@brc.hu (S.V.); deli.maria@brc.hu (M.A.D.); der.andras@brc.hu (A.D.)
[2] Doctoral School of Multidisciplinary Medical Sciences, University of Szeged, Dóm tér 9, Szeged, H-6720, Hungary
[3] Doctoral School of Biology, University of Szeged, Közép fasor 52, Szeged, H-6726, Hungary
* Correspondence: der.andras@brc.hu
# Authors contributed equally to the manuscript

Methods

Barrier integrity permeability studies

Directly after the 30 min protein passage assay a short, 10 min permeability experiment was performed on the control cells, which only received the 0.1% BSA-RH without the spike protein treatment. Since there was no difference between the TEER values of the cells treated with spike protein or the 0.1% BSA-RH only, we only performed permeability assay with fluorescent marker molecules for the control cells. Flux of two fluorescent dyes was investigated: FITC-dextran 4.4 kDa (FD4, 100 μg/ml) for the BBB model and sodium fluorescein (SF, 10 μg/ml) for the Caco-2 cells in 0.1%BSA-RH solution similarly as described previously [1; 2]. Permeability of cell-free inserts was also measured and was used to calculate the cellular monolayer permeability coefficient (Pe) [1].

Supplementary Figures

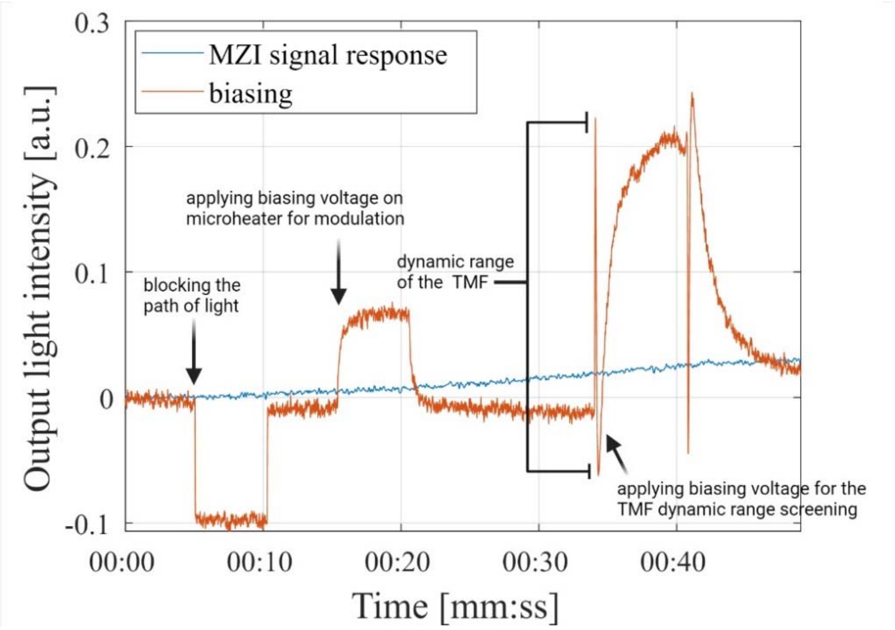

Figure S1. The demonstration of the bias point calibration process with the Mach-Zehnder interferometer (MZI) output intensity values during the biasing process and during the measurement. For the calibration, first the initial light intensity value was set at a baseline. Then the incoming light path was blocked for a short period to evaluate the range of the initial intensity. Biasing DC voltage was applied on the microheater with a given amplitude for phase modulating one arm of the interferometer. The voltage amplitude was set to reach and lock the system at the ideal inflexion point on the sinusoidal transmission function (TMF) of the MZI providing desired working conditions. Finally, the same process was performed by applying the biasing voltage with higher amplitude, thus screening the dynamic range of the sensor, where the minimum and maximum output ontensities could be seen, thus enabling the checking of the heating voltage amplitude set previously. The figure was created with Biorender.com.

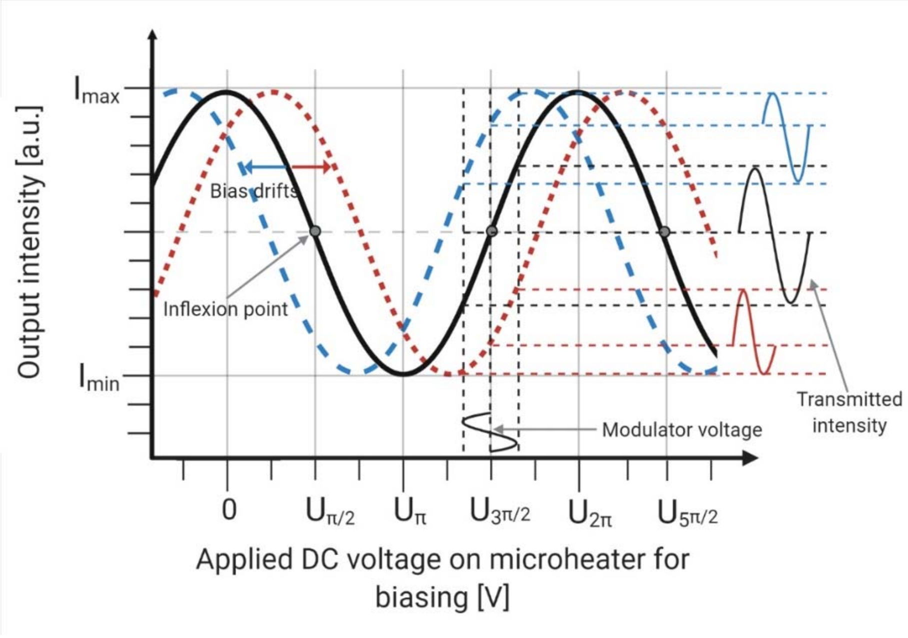

Figure S2. Sinusoidal transmission function curve of the Mach-Zehnder interferometer with the highlighted, ideal inflexion points for biasing on it in a function of the applied voltage, applied for the thermal phase shifting ($U_{\pi/2}, U_{\pi}, U_{3\pi/2}$ etc.). As it can be seen, without appropriate bias point adjustment a drift in the MZI transfer function results in a change of the optical modulated signal with less effective amplitude modulation and significant distortions. The figure was created with BioRender.com

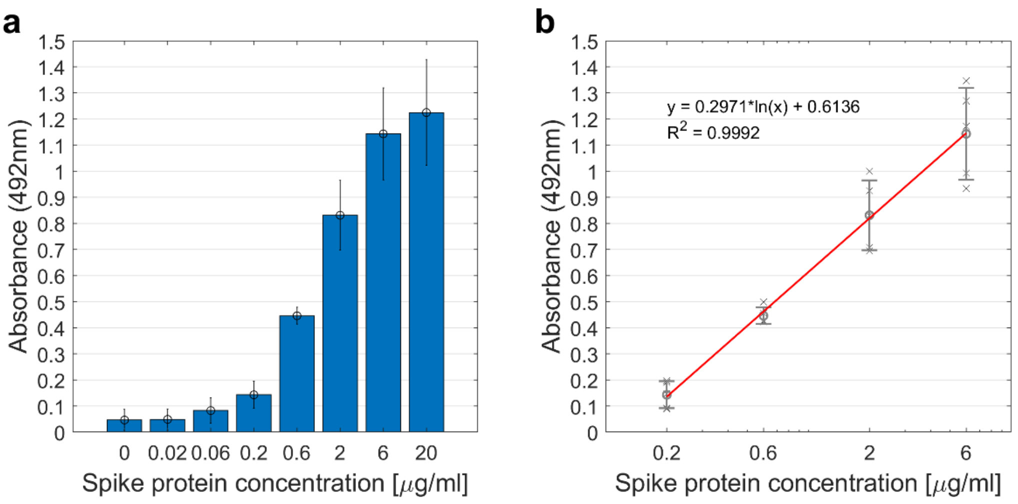

Figure S3. ELISA immunoassay calibration: assay results of the calibrator samples **(a)**, and the fitted calibration curve **(b)** for the evaluation of the spike protein solution concentrations of proteins, passed through the cell culture models.

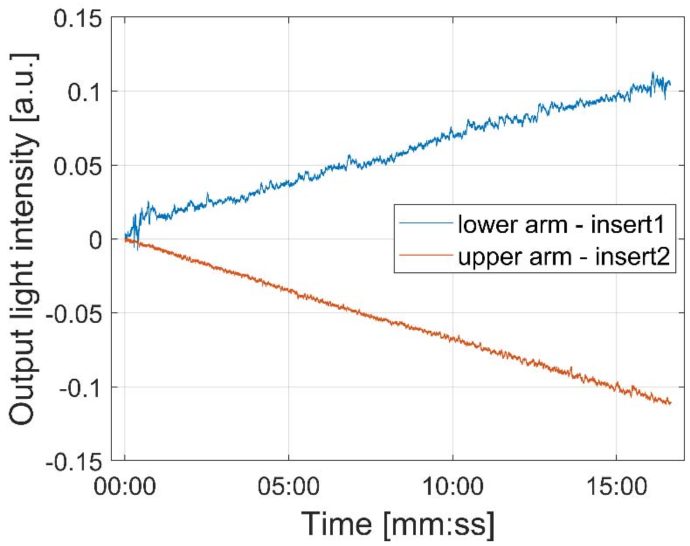

Figure S4. Demonstration of the intrinsic feature of such interferometric detection. During the target Spike protein detection from fluid sample, an increasing, or decreasing sensor response with equal slope was obtained depending on which branch of the interferometer was in use as a measuring arm. In this case fluid sample collected from inestinal barrier (Caco-2 cells) model assay insert was investigated.

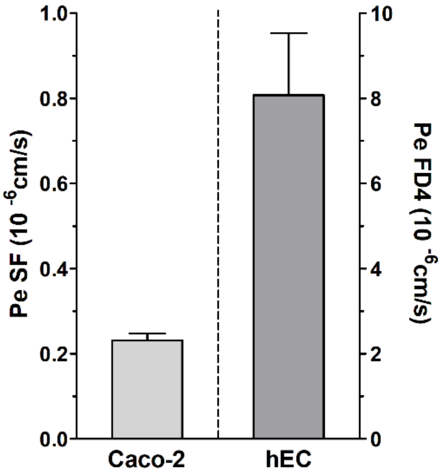

Figure S5. Permeability of Caco-2 monolayers, a culture model of the intestinal barrier, and the human endothelial cell (hEC) – brain pericyte blood-brain barrier co-culture model. Assay was performed on control groups, after receiving 0.1%BSA-RH for 30 min. Permeability for fluorescent marker molecules sodium fluorescein (SF, 376 Da; 10 μg/ml) and FITC-dextran 4.4 kDa (FD4, 100 μg/ml) for 10 min was performed to test barrier integrity. Values presented are means ± SEM, n = 3-4. Permeability is expressed as endothelial/epithelial permeability coefficient (P_e). Both cell types expressed permeability values similar to our previous observations [3, 4].

References

1. Hülper, P., Veszelka, S., Walter, F. R., Wolburg, H., Fallier-Becker, P., Piontek, J., Blasig, I. E., Lakomek, M., Kugler, W., & Deli, M. A. Acute effects of short-chain alkylglycerols on blood-brain barrier properties of cultured brain endothelial cells. *Br J Pharmacol* **2013**, *169*, 1561–1573, https://doi.org/10.1111/bph.12218
2. Bocsik, A., Gróf, I., Kiss, L., Ötvös, F., Zsíros, O., Daruka, L., Fülöp, L., Vastag, M., Kittel, Á., Imre, N., Martinek, T. A., Pál, C., Szabó-Révész, P., & Deli, M. A. Dual Action of the PN159/KLAL/MAP Peptide: Increase of Drug Penetration across Caco-2 Intestinal Barrier Model by Modulation of Tight Junctions and Plasma Membrane Permeability. *Pharmaceutics* **2019**, *11*, 73, https://doi.org/10.3390/pharmaceutics11020073
3. Hellinger, E., Veszelka, S., Tóth, A. E., Walter, F., Kittel, A., Bakk, M. L., Tihanyi, K., Háda, V., Nakagawa, S., Duy, T. D., Niwa, M., Deli, M. A., & Vastag, M. Comparison of brain capillary endothelial cell-based and epithelial (MDCK-MDR1, Caco-2, and VB-Caco-2) cell-based surrogate blood-brain barrier penetration models. *Eur J Pharm Biopharm* **2012**, *82*, 340–351, https://doi.org/10.1016/j.ejpb.2012.07.020
4. Santa-Maria, A. R., Walter, F. R., Valkai, S., Brás, A. R., Mészáros, M., Kincses, A., Klepe, A., Gaspar, D., Castanho, M., Zimányi, L., Dér, A., & Deli, M. A. Lidocaine turns the surface charge of biological membranes more positive and changes the permeability of blood-brain barrier culture models. *Biochim Biophys Acta Biomembr* **2019**, *1861*, 1579–1591, https://doi.org/10.1016/j.bbamem.2019.07.008
